# Key Factors Influencing Low-Carbon Behaviors of Staff in Star-Rated Hotels—An Empirical Study of Eastern China

**DOI:** 10.3390/ijerph17218222

**Published:** 2020-11-06

**Authors:** Jie Li, Peng Mao, Hui Liu, Jiawei Wei, Hongyang Li, Jingfeng Yuan

**Affiliations:** 1 School of Civil and Transportation Engineering, Shenzhen University, Shenzhen 518060, China; lijie20181@email.szu.edu.cn; 2 College of Civil Engineering, Nanjing Forestry University, Nanjing 210037, China; maopeng@njfu.edu.cn (P.M.); liuhui@njfu.edu.cn (H.L.); 3 Research Institute of Zhongjiao Sihang, Guangzhou 510220, China; wjiawei1@cccc4.com; 4 Business School, Hohai University, Nanjing 211100, China; 5 School of Civil Engineering, Southeast University, Nanjing 210096, China; jingfeng-yuan@seu.edu.cn

**Keywords:** low-carbon behaviors, hotel staff, influencing factors, star-rated hotels, targeted strategies

## Abstract

To guide sustainable development in the hospitality industry requires hotel staff engagement, so what causes and how to facilitate the implementation of low-carbon behaviors should be high priorities. However, most prior studies focused on hotel guest behavior or discussed, on an individual level, the psychological aspects of the factors of the low-carbon behavior of either managers or employees. Therefore, this research aims to examine the effect of influencing factors inside and outside of the hotel context on hotel staff’s low-carbon behaviors in star-rated hotels. A set of influencing factors were identified by using literature retrieval, ground theory and in-depth interviews. Structural equation modelling was then applied with 440 valid questionnaires collected from representative star-rated hotels in Eastern China. The results revealed that low-carbon managerial activities, strategic orientation, social norms, and perceived behavior control were four key factors affecting the low-carbon behavior adoption of staff from star-rated hotels. Among them, low-carbon managerial activities were found to be the strongest factor affecting hotel staff’s low-carbon behaviors. Consumer attitude, however, exerted no significant impact. Targeted strategies were finally proposed for the improvement of hotel staff’s low-carbon behavior from the perspectives of hoteliers and governments. This study contributes to the generation mechanism of low-carbon behavior among staff and, in practice, towards behavioral improvement by providing comprehensive insights about the attribution of factors belonging to multiple dimensions related to the low-carbon behavior of staff in the hotel industry.

## 1. Introduction

Global warming opens Pandora’s box—with adverse impacts on the environment, aggravated by human behaviors, without effective intervention. The lodging industry, involving various anthropogenic activities, poses great environmental pressure to ecosystems [1]. Hotels, as the primary units of accommodation, have witnessed an imposing growth in tourism in recent years. However, they have also become major energy-intensive end users in many countries due to the heavy dependence on the supply of energy, accounting for a significant proportion (around 20%) of carbon dioxide emissions in the tourism sector on a global scale [2,3]. Meanwhile, the domestic situation regarding this is equally dire in China. Statistically, hotels were the second highest energy-consuming buildings in Beijing, the capital of China, after shopping malls, among commercial buildings [4]. A similar situation was reported in the research of Xing et al., 2015 [5] in which hotel buildings consumed higher energy than any other types of non-residential buildings in Tianjin, China. Nowadays, hotels in China suffer the problems of a high cost input, high energy consumption, and high environmental pollution (so-called 3H problems) during their daily operations due to 24/7 arrangements, superior facilities/functions, and a free reign on energy use in guests’ rooms [6]. In particular, many sizable star-rated hotels providing higher-level living comfort and service quality result in more energy/water use and larger amounts of greenhouse gas emissions. They are thus chief contributors to environmental issues in the lodging industry [6,7]. In this context, star-rated hotels face an imperative shift towards low-carbon development. It is therefore significant to promote effective low-carbon practice in star-rated hotels.

Over the last few years, the hotel sector in China has started to implement a wide spectrum of low-carbon practices to comply with sustainable demand across the whole of society, such as sustainable management regulations, recycling products/facilities, and green training [8,9,10]. Nevertheless, the situation of CO_2_ emissions in hotels seems not to have been mitigated much and, instead, an average annual increase of 3.2% in China was witnessed [11]. Hotel staff, as major actors who provide accommodation services, are endeavoring to solve such issues. Particularly, the low-carbon strategies are proposed by those at the top management level, aiming to meet industrial or societal expectations and, accordingly, internal employees’ low-carbon engagement needs to remain consistent with their organizations [12]. It seems that they have more control than outside guests over the low-carbon performance of hotels through their own behavior change. However, some hotel staff, either hoteliers or employees, exhibited non-low-carbon behaviors in practice [13]. Therefore, it is especially important to research the factors underlying hotel staff’s low-carbon behaviors so as to enable their effective low-carbon work engagement in star-rated hotels.

The previous literature regarding low-carbon behavior in the hospitality industry mainly focused on the guest side, including drivers of guests’ intentions to behave pro-environmentally or guest loyalty in relation to visiting green hotels, guest perception of low-carbon practice in hotels, and so forth [2,14]. Compared with this, the factors that influence hotel staff’s low-carbon behaviors in the workplace have been relatively neglected [12]. Although there are existing empirical studies on what causes the pro-environmental behavior of hotel employees, the majority discussed these causes on individual levels, such as environmental beliefs, personal attitudes, intrinsic motivations, environmental knowledge, environmental awareness, environmental concern, work engagement, etc. [15,16,17,18]. There is still a relative paucity of research on the factors influencing hotel staff’s low-carbon behaviors by taking into consideration the multiple dimensions inside and outside of the hotel context, leading to limited contributions to the theoretical mechanisms of factor effects on staff’s low-carbon behaviors, as well as to practical guidance for star-rated hotels becoming more green.

Based on such a research gap, this research aims to identify the key influencing factors of staff’s low-carbon behaviors in star-rated hotels in the context of Eastern China. Three specific sub-motivations are proposed: (1) What are the key factors affecting staff’s low-carbon behavior implementation? (2) How do the identified factors affect staff’s low-carbon behaviors in star-rated hotels differently? (3) What strategies could be proposed to improve staff’s low-carbon behaviors in order to boost star-rated hotels’ efforts to become much greener? The findings from this research will contribute to compensating for the limitation of studying influencing factors on staff’s low-carbon behaviors in star-rated hotels from both inside and outside of the hotel context, deepening our understanding of behavior generation mechanisms in the lodging industry. Moreover, this study provides valuable implications for how to guide hotel staff towards low-carbon behaviors by incorporating key influencing factors into effective strategies for hoteliers and the authorities, which assist star-rated hotels in China to achieve environmentally friendly industrial development in order to contribute to the long-term sustainability of society.

## 2. Literature Review

### 2.1. Previous Research on Hotel Low-Carbon Behavior

Low-carbon behaviors were defined by Stern (2000) as behaviors affecting the utility of substances or energy positively, and those able to alter the structure and dynamics of an ecosystem/biosphere positively [19]. Hence, the deep retrieval of hotel actions, such as “low carbon practice”, “sustainable management”, “corporate sustainability”, etc., is needed when it comes to low-carbon behavior in the lodging industry. Some scholars highlighted how a hotel sector put low carbon into practice from the perspective of eco-technology adoption, including integrated CO_2_ systems, renewable energy alternatives, HVAC (Heating, Ventilation and Air Conditioning) system renovation, etc. [20,21,22]. Nevertheless, the environmental performance of hotels, as a typical, traditional service, seems more sensitive in contrast to how hotel stakeholders behave. To date, existing studies have focused primarily on consumer behavior in hotels and presented different antecedents of consumer behavioral intentions towards green hotel visits, willingness to pay, check-in satisfaction and loyalty, or the reuse activities of hotel products [2,17,23,24,25].

Furthermore, there has been an intensification of concern for environmental protection and green practice in hotels, with increasing studies providing insights about the operator experience in the hospitality industry. For instance, Stefanica et al., (2020) identified that the ecological purchase behaviors of hotel managers were positively influenced by environmental attitudes and economic benefits in an investigation covering 92 hotels in Romania [26]. Volpi and Paulino (2018) argued that the importance of the lodging service that hotels provided could not be denied regarding the environmental requirements of sustainable tourism; however, in their research, the statistics were only partially related to the environmental performance of the sector [27]. Moreover, Mar and Rodríguez (2011) conducted an empirical analysis to highlight the roles of certified management systems in the hotel business and found organizational behavior differences between the implementation of management systems in the Spanish hospitality industry [28]. Nonetheless, most relevant studies partly considered how hotels implemented low-carbon practice on one single level of operation. Although some research papers concluded that both the sustainable strategies of top-level management and the organizational work practices of lower managers and front-line employees were integral parts of corporate sustainability in the hotel industry [29], they were merely concerned about behavior translation modes, without exploring the underlying determinants of different staff behaviors. This research is thus targeted at identifying which key factors trigger low-carbon behaviors, embodying how staff at different levels act in an eco-friendly manner in the lodging industry.

The current literature shows no evidence that there is a unified definition of the low-carbon behavior of staff in hotels, but scholars have researched different aspects that they believe to be of relevance from their own points of view. For instance, Ann and Pearce (2013) used the case study method to identify and discuss low-carbon design strategies in two luxury hotels in America [30]. Hsu et al., (2014) established an evaluation model of suppliers’ carbon and energy management performance to research how to select low-carbon suppliers based on the hotel industry [31]. Liu and Pan (2016) pointed out that hotel employees should try their best to minimize the use of facilities that consume a lot of power and encourage guests to reduce the use of disposable cleaning products [32]. As low-carbon behavior belongs to pro-environmental behavior in the domain of environmental sociology and it derives from corporate social responsibility, which shows the voluntary corporate commitment to promoting social and environmental goals when it comes to an enterprise’s pro-environmental behavior [33], this paper defined the low-carbon behavior of staff in star-rated hotels as daily anthropogenic activities in relation to hotel staff at different levels, from upper management to middle- and lower-level employees. Their behaviors were grouped into five categories according to the literature retrieval results and hotel sector functions, as shown in Table 1.

### 2.2. Previous Research on Factors Influencing Low-Carbon Behavior in Hotels

In the current literature, there exist many different studies exploring factors affecting low-carbon behaviors in the hospitality industry. Verma and Chandra (2018) extended the model of the theory of planned behavior, including reflectiveness and conscientiousness, to predict young Indian consumers’ green hotel visit intentions [34]. Chuah et al., (2020) explained the psychological mechanisms and boundary conditions of how perceived corporate social responsibility–brand fit affected sustainable customer engagement behavior [26]. Merli et al., (2019) tested the relationship between guest perceived hotel green practice performance and their behavioral intentions in ecolabel-awarded hotels, concluding that staying at green hotels leads guests to develop loyalty toward them [2]. Moreover, research by Gössling et al., (2019) showed that various factors influenced the pro-environmental behavior of hotel tourists, such as nationality, age, length of stay, and comprehensive message designs [35]. However, these scholars paid more attention to what triggered the low-carbon behavior of hotel guests rather than on the hotel service side of the hospitality industry. Thus, there is still sufficiently large scope to research the influencing factors of hotel staff’s low-carbon behavior.

The environmental behavior of guests, on the one hand, and low-carbon hotel marketing, on the other hand, have both urged the accommodation sector to reconcile the conventional conflicts between economic benefits and environmental quality [36]. Hence, researchers have also been building upon the view of hotel service and their concerns about the guest perception of hotels’ low-carbon practice has recently grown [37,38]. However, they mainly explored how contributors affected low-carbon behaviors in hotels from individual guests’ experiences. Additionally, Fatoki (2019) pointed out that leadership mechanisms and workplace support could nurture hotel employees’ pro-environmental behaviors [12]. Molina et al. (2015) deemed that that the ability of employees to utilize their knowledge or skills in low-carbon practice reflected the capability of green quality management in the hotel industry [39]. Wong and Kim (2020) confirmed that hotels’ corporate social responsibility, as perceived by internal staff, made a difference to sustainability and responsible management in the lodging industry [40]. In another piece of research by Osman et al., (2020), they investigated the impact of job insecurity and work engagement on hotel employees’ non-green behaviors and found that job insecurity eroded work engagement and exacerbated non-green behaviors [18]. Nonetheless, the above relevant studies were conducted on individual levels, mostly in relation to psychological aspects, in explorations of the factors influencing the low-carbon behavior of either managers or employees in hotels.

There are some other studies discussing non-staff related factors. Dube and Nhamo (2020) pointed that policy elements like municipal bylaws could guide the ideals of green and sustainable development for hotel construction activities [41]. Similarly, Mohammad et al., (2021) believed that policymakers, as key stakeholders, should make an attempt at adjusting punitive economic sanctions in time to provide hoteliers with access to the latest modern energy conservation technologies [42]. Design issues are regarded as among the mitigating factors that influence hotel staff to adopt low-carbon behavior. For instance, the architectural design of thatching may make it difficult to install renewable energy products in the operation stage of the hospitality sector [42]. Overall, empirical studies provide a holistic view of the factors inside and outside of hotels that affect low-carbon behavior among staff in the lodging industry and show that research in this area is still limited.

## 3. Factor Identification and Hypotheses Development

### 3.1. Literature Retrieval Process

Retrieving the existing studies is a key step for factor identification. In order to systematically and precisely collect the variables, inducing low-carbon behavior among staff in star-rated hotels, Web of Science (WoS) was primarily chosen for literature retrieval as it was judged to be one of the most comprehensive and dependable sources, covering not only the domains of natural science and engineering, but also social science, arts and humanities [43,44]. An advanced search provided by the WoS core collection database was conducted with the following retrieval codes: TS = ((hotel* OR lodging* OR hospitality) AND ((green OR low-carbon OR low carbon OR sustainabl*) AND (behavio* OR practic* OR manag* OR operat*))). In total, 1923 relevant articles published in English since 2010 were initially retrieved from WoS. These papers were further filtered according to research orientations and the top 50% of cited journals in the database. Other well-known databases, including ScienceDirect, Google Scholar and Scopus were then searched to update and supplement the search results. Finally, 657 directly related articles were critically reviewed from *Sustainability* (99), *International Journal of Contemporary Hospitality Management* (84), *International Journal of Hospitality Management* (82), *Journal of Sustainable Tourism* (65), *Journal of Cleaner Production* (36), and *Tourism Management* (35), to name but a few.

### 3.2. Theoretical Background

Behavior tends to be triggered by certain stimuli, also called drivers of behavior [45], so it is critical to understand what induces low-carbon behavior among staff in star-rated hotels. There are many explorations on the influencing factors of people’s low-carbon behaviors, and such studies mainly used the theory of planned behavior (TPB) to illustrate the relationship between the subjects’ behaviors and psychology-related factors [34,46]. However, the explored TPB model did not include all triggers relevant to pro-environmental behavior, only those on an individual level. When it comes to corporate behavior, which is always dominated by top-level management, especially in energy-intensive industries like tourism, hospitality and transport, many concerns are increasingly being raised on the altruistic effort that corporates make towards social and environmental benefits, and these studies mostly come from the theory of corporate social responsibility (TCSR) [47,48]. Concerning that the studied hotel staff include both top management and front-line employees, TPB and TCSR are merged in this research. Moreover, there is a general consensus that social context strongly affects people’s actions [49,50]. Particularly in classical problems of social theory, those regarding behaviors influenced by social relationships or stakeholder interactions, are trying to be solved by researchers [51]. Therefore, from the perspective of embedding the behavior of star-rated hotel staff in a social environment, it is also necessary to introduce influencing factors from outside hotels. This point and the above two theories complement each other, and as such construct the theoretical basis of this research.

### 3.3. Exploratory Identification Results

Orientated by the theoretical basis of this research, the variables triggering low-carbon behavior among staff in the hotel industry were collected, but they are variegated and dispersed in the existing literature (shown in the third column in Table 2). Therefore, this paper employed grounded theory, which was regarded as creating an “explanation of action” [52] to identify six influencing factors of relevance for our research aim: strategic orientation, low-carbon managerial activities, personal norms, perceived behavior control, social norms, and consumer attitude. In order to ensure the systematic and comprehensive factor identification in an exploratory and inductive process, twelve experts from different levels of star-rated hotels in Eastern China were interviewed in-depth and face-to-face for further confirmation between July and August 2018. Purposive sampling was administered for interviewee selection according to their potential contributions to the topics we expected to broach. All chosen interviewees had at least five years of experience in the lodging industry, including four upper managers and eight middle-level managers. Among them, three were from five-star hotels, four from four-star hotels, and five from three-star hotels. Under the data saturation principle followed in the study of [53], no updated information was gained from the interview with the twelfth interviewee, indicating that data were saturated after twelve interviews with no more interviewees needed after this point. According to the above research process, a set of six influencing factors of low-carbon staff behavior in star-rated hotels was finally identified (Table 2).

### 3.4. Hypotheses Proposed

#### 3.4.1. Strategic Orientation

In the lodging industry, it is not only the “right thing to do”, but also “the smart thing to do” for hotel enterprises to attract customers and dominate marketplaces [54]. Hence, understanding strategic orientation is a critical marketing strategy for hotels, as it creates proper behaviors that enable the continuous superior performance of a hotel business [55,56]. Many high-end hotels have gradually realized the need to focus not only on profit promotion, but also on decision making that is ethically and socially acceptable in relation to the environment involved [57], making strategic orientation largely mirror cooperate social responsibility. Thus, the first hypothesis is formulated as follows:

**Hypothesis** **1.** *Strategic orientation exerts a direct positive effect on staff’s low-carbon behaviors in star-rated hotels.*

#### 3.4.2. Low-Carbon Managerial Activities

Hotel managers play an important role in implementing appropriate sustainability practices [58]. In the current literature, the dimension of study of the environmental management practice of hotels has expanded to diversity based on a plurality of management activities, such as communication and education, marketing activities, and organizational and operational practices [59]. Additionally, as proposed in the research of [60], low-carbon managerial actions, e.g., improvements in facilities or materials, tax incentives, and other energy-saving programs, are of great benefit during the operation process of hotel enterprises. Accordingly, the following hypothesis is then proposed:

**Hypothesis** **2.** *Low-carbon managerial activities exert a direct positive effect on staff’s low-carbon behaviors in star-rated hotels.*

#### 3.4.3. Personal Norms

Personal norms, as critical determinants of pro-environmental behaviors, refer to the expectations people hold for themselves [19,61]. Gao et al., (2017) proved that these norms derived from moral responsibility or the obligation to perform/refrain from specific behaviors [48]. In other words, people behave in a way that they believe is morally right; for instance, employees tend to be willing to follow energy-saving actions regulated by hotels. Harland et al., (2007) also asserted that pro-environmental behaviors would be encouraged when people showed moral obligation towards environmental issues [62]. Thus, our model proposes the assumption that personal norms influence low-carbon behavior in star-rated hotels:

**Hypothesis** **3.** *Personal norms exert a direct positive effect on low-carbon behavior among staff in star-rated hotels.*

#### 3.4.4. Perceived Behavior Control

Perceived behavior control represents the extent of volition people perceive that they have and whether they feel they can actually perform an action [63]. This means the more time, knowledge, energy, skills, resources and opportunities someone believes they possess, the fewer obstacles they may expect in carrying out the behavior [64]. As Kollmuss and Agyeman (2002) indicated, people took environmental protection actions only if they mastered relevant knowledge of environmental issues and behaviors that may cause eco-problems [65]. Similarly, Donald et al. (2014) found that if individuals had higher levels of knowledge and skills to save energy, they would focus on energy conservation in their daily life [66]. This evidence is therefore tested with the following hypothesis:

**Hypothesis** **4.** *Perceived behavior control exerts a direct positive effect on staff’s low-carbon behaviors in star-rated hotels.*

#### 3.4.5. Social Norms

Social norms represent the restraint effect of social pressure on each individual, featured as mandatory [67]. There is currently a large amount of evidence that social norms nudge people towards a wide range of environmental choices and behaviors [68,69]. It was found that the social pressure caused by social norms had a great impact on behaviors of energy use and personal low-carbon practice [70]. Another study by Nolan et al., (2008) showed that people began to increase towel reuse in guest rooms after being informed about the reuse condition by others [71]. Social norms in the lodging sector normally cover hotel rules and regulations, public environmental supervision, peer competitiveness from other hotels, the values that social groups and members acknowledge, etc. As people do not act as in isolation, they are likely to be easily affected by normative influences and surrounding contexts [72]. Thus, the paper tests the following hypothesis:

**Hypothesis** **5.** *Social norms exert a direct positive effect on low-carbon behavior among staff in star-rated hotels.*

#### 3.4.6. Consumer Attitude

Attitude is how an individual feels about their behaviors (e.g., positive or negative) [73]. Star-rated hotels place consumers at their center since their attitudes are a pivotal factor for hotel survival, which is strongly associated with repeat sales, positive word of mouth, and guest loyalty [74]. Increasing attention being paid to the environment and sustainability by consumers could lead hoteliers to upgrade their business by adopting low-carbon hospitality management [2]. Namely, if a star-rated hotel wants to gain and keep guests with positive attitudes towards low-carbon operation, hoteliers will undoubtedly make great efforts to conduct a wide spectrum of low-carbon practices, such as green product procurement, energy and water savings, and waste recycling, to meet the demand of sustainability-sensitive guest segments [34,75,76,77]. Hence, the following hypothesis is proposed:

**Hypothesis** **6.** *Consumer attitude exerts a direct positive effect on staff’s low-carbon behaviors in star-rated hotels.*

**Figure 1:**
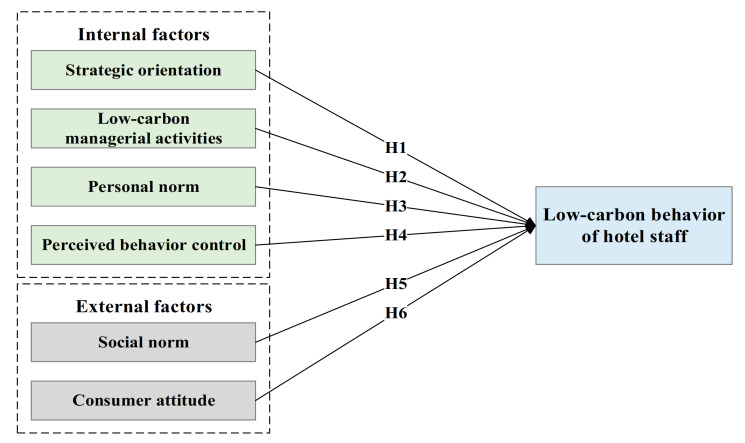
Theoretical model and hypotheses. (Note: H stands for hypothesis).

Based on the above hypotheses, the theoretical model to be tested in our empirical study was constructed as shown in Figure 1.

## 4. Methodology

### 4.1. Questionnaire Design

After a series of preliminary research tasks and a pilot study, behavioral and factor-related questions were designed based on five of the low-carbon behaviors of hotel staff [30,31,32] and six of the identified influencing factors [8,78,79,80,81,82]. A pilot study was conducted in Nanjing, one of the cities in the targeted study area, and fifty staff volunteers in star-rated hotels took part in it in August 2018. Moreover, to further ensure the content validity of the designed questionnaire, it was also reviewed by experts in the abovementioned expert interviews. Based on these results, questions that were unclear or problematic were revised.

The final questionnaire covers three main sections: (1) the socio-demographics of respondents, as well as star ratings of the hotels they work for and some basic hotel information; (2) the degree of impact of the six factors influencing staff’s low-carbon behaviors in star-rated hotels; and (3) the actual implementation of low-carbon behavior among staff for star-rated hotels. Notably, the behaviors in this research signified self-reported behaviors regarding low-carbon design, low-carbon procurement, low-carbon decision making, low-carbon operation and low-carbon execution, which are usually adopted as proxies of actual human activities due to their high efficiency and low cost [83]. Twenty-nine items were measured, depending on seven latent variables: three for strategic orientation (SO), six for low-carbon managerial activities (LCM), three for personal norms (PN), three for perceived behavior control (PBC), six for social norms (SN), three for consumer attitude (CA), and five for low-carbon behavior (LCB), as shown in Table 3. All items in Section 2 and Section 3 of the questionnaire were rated on a seven-point Likert scale from which respondents were asked to rate from 1 (“strong disagreement”) to 7 (“strong agreement”) how well each measurement described their experience and practice with these factors.

### 4.2. Data Collection

**Figure 2:**
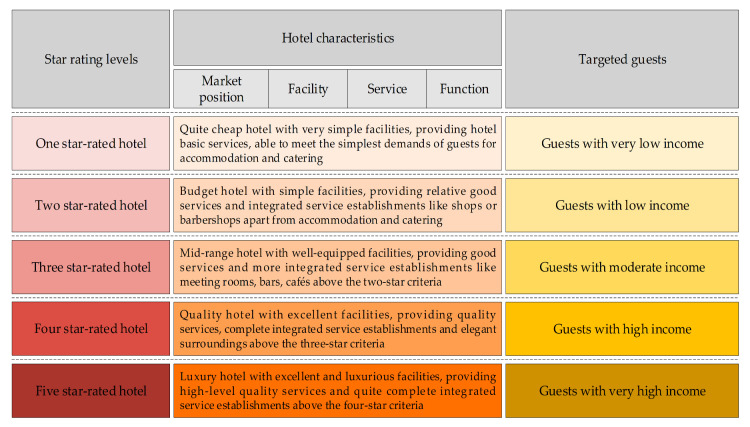
Criteria for classifying hotels with different stars.

According to the Star Rating and Evaluation of Tourist Hotels (GB/T14308-2010), released by the National Tourism Administration in China, a five-star rating system was applied to evaluate the star ratings of different hotels according to hotel architectural scales, service equipment, service quality, management levels, etc. (Figure 2). Namely, hotels could be classified into five rating levels from one star-rated hotels to five star-rated hotels, which means that the more stars a hotel has, the higher rating level it belongs to. It was found in the first-stage field investigation that hotels rated as having no more than three stars were not commonly mature in regard to their low-carbon practice: for example, their employees had a weak low-carbon awareness, they had less advanced energy-saving facilities and equipment, less green publicity, and so forth. Therefore, considering the validity of answers given by hotel staff in the survey, the hotels rated as having three and more stars were selected as star-rated hotels in this research.

**Figure 3:**
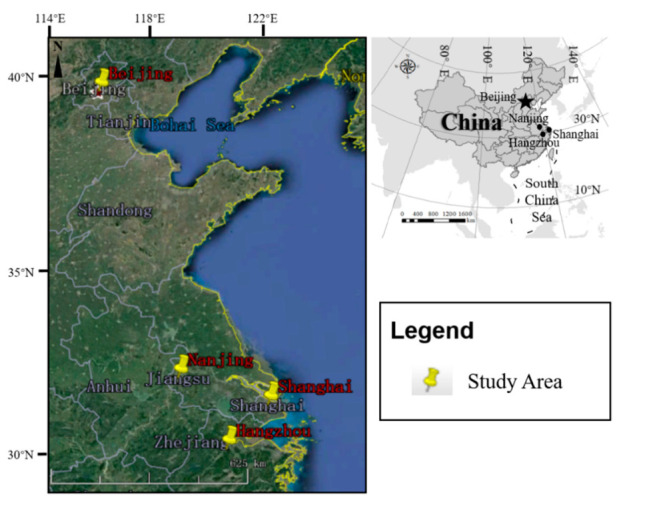
Study areas of the questionnaire survey.

The formal questionnaire survey was administered from September to October 2018. Questionnaires were distributed to staff (including front-line staff members and managers) from star-rated hotels in typical metropolises (municipalities or provincial capitals) of Eastern China, including Beijing, Shanghai, Hangzhou, and Nanjing (Figure 3). The per capita carbon emissions in these megacities are usually higher than core cities of other nations like London, Singapore, and Tokyo [84,85], needing more attention when developing low-carbon strategies.

It is worth mentioning that the connotation of low-carbon behavior among staff in our research is rather different from what is generally understood, especially in the fields of medical health. Instead, it refers to what actions hotel staff could take to lead the green, sustainable and low-carbon development of star-rated hotels, which has nothing to do with any ethical issues related to the human body. Therefore, we must stress that the present research objective is not targeted at individual behavior that involves human subjects, human material, human tissues or human data. Another thing that we must note is that, prior to the formal questionnaire survey in the investigated star-rated hotels, we communicated adequately with the hotels’ direct supervisors to confirm whether data collection would be permitted in their hotels. Then, all the staff from the investigated star-rated hotels were informed of the study purpose and intent with the coordination of the hotels’ direct supervisors. Moreover, they were previously promised that their answers would not be leaked out to any others and that their experience would be fully anonymous with no private information identifying them included. After such a procedure, which played the same role as an ethical statement, a total of 500 hotel staff members agreed and volunteered to participate in the survey. Finally, there were 440 valid responses returned both offline 75.5% (332) and online 24.5% (108), resulting in an effective response rate of 88.0%. This questionnaire survey gave priority to offline collection and was supplemented with online collection, because—helped by hotels’ direct supervisors—we got in in direct touch with some front-line staff members who agreed to participate in our survey through the internet. The socio-demographic information of the respondents is shown in Table 4.

### 4.3. Data Analysis

Structural equation modeling (SEM) is a method widely used in the domain of behavioral science [86,87,88,89]. It can not only estimate a series of independent multiple regression equations simultaneously, but also incorporate latent variables by taking measurement errors into account. Superior to multiple regression analysis, SEM is robust to measurement errors and model misspecification [90]. Hence, it was selected in this research to analyze the impact of influencing factors on staff’s low-carbon behaviors in star-rated hotels. The maximum likelihood method was applied in SEM, for the use of which the normality assumption should not be severely violated [91]. Based on such a precondition, descriptive statistics analysis and reliability analysis were initially conducted assisted by IBM SPSS Statistics (V.23) (IBM, Armonk, NY, USA). IBM SPSS Amos (V.24) (IBM, Armonk, NY, USA) was then employed to build and modify models and conduct confirmatory factor analysis.

## 5. Results

### 5.1. Descriptive Statistics

The mean values (M) and standard deviations (SD) of construct items are obtained as listed in Table 5. All M-values are greater than five, ranging from the lowest at 5.63 (CA3, referring to consumer comments) to the highest at 6.45 (SN1, referring to macro-level market policies), indicating an overall positive response to the considered constructs. The normality assumption test is the premise of the utilization of the maximum likelihood method in confirmatory factor analysis, and severe abnormally distributed samples, suggesting absolute skewness (*Sk*) values greater than three and absolute kurtosis (*K*) values greater than 10 [92], are not acceptable. As shown in Table 5, the skewness values and kurtosis values of construct items were mostly close to zero, with no value falling out of the above constraint interval (i.e., *Sk* ∈ (−3, 3) ∩ *K* ∈ (−10, 10)), which implied that the sample data were generally normally distributed. Thus, the maximum likelihood method could be used for estimation in further analyses.

### 5.2. Measurement Model Evaluation

#### 5.2.1. Reliability Analysis

To test the internal consistency of the constructs, Cronbach’s α (0–1) is normally used, based on the average inter-item correlation. Reliability is acceptable when the α value is more than 0.7 [93]. Cronbach’s α of each construct and the overall sample were analyzed. All α values are more than 0.8 (most of them >0.9), indicating the high reliability of the collected data (Table 6). Furthermore, composite reliability (CR) among constructs ranges from 0.826 to 0.960, all values of which exceed the standard minimum limit of 0.7, suggesting stronger reliability than Cronbach’s alpha estimation [94].

#### 5.2.2. Validity Analysis

Considering that the preliminary factor identification process is exploratory and inductive, exploratory factor analysis (EFA) is needed to provide strong evidence in order to verify the high-quality questionnaire development in this research. Therefore, pre-Principal Component Analysis (PCA) was used to carry out EFA. The rotated component matrix and extraction details are both shown in Table 7. The items were finally classified into six corresponding factors according to the criterion of the factor loading’s absolute value larger than 0.5 [95]. For better visualization, the values below such a threshold are excluded in Table 7. Notably, the classification results in Table 7 fully correspond to the previous identification results of influencing factors, illustrating that the factor identification and questionnaire design are rigorous and convincing. Overall, the sample data meet the requirements for SEM analysis.

The goodness-of-fit indices of the initial measurement model are indicated in Table 8, all of which met the acceptable thresholds of model fitness adapted from the studies of Leung and Chan (2007), as well as Qureshi and Kang (2015) [96,97]. This ensures the appropriateness of the following confirmatory factor analysis (CFA) for the validity test. Through the CFA, the measurement items with standardized factor loadings (FL) less than 0.5 need to be eliminated [98]. None of the items were deleted in this research, since all their standardized factor loadings exceeded the recognized value (*p* < 0.001). Apart from the factor loading test, converged validity and discriminant validity were then tested. For the constructs in Table 8, all the average variances extracted (AVE) passed the cut-off value of 0.5 [99], with a minimum of 0.614 and a maximum of 0.888, which indicated the strong convergent validity of items for the same construct.

The discriminant validity was subsequently tested, in which the square root of the AVE for a given construct was compared with the correlations between such a construct and all others [100]. Table 9 shows that the square root of each AVE (diagonal elements) is greater than the related inter-construct correlations (off-diagonal elements) in the corresponding rows and columns, indicating adequate discriminant validity for all the constructs. From the above, the measurement model evaluation shows high reliability and validity of constructs and corresponding items for further assessment of the structural model.

### 5.3. Structural Model Assessment

#### 5.3.1. Results of Model Modification and Test

**Figure 4:**
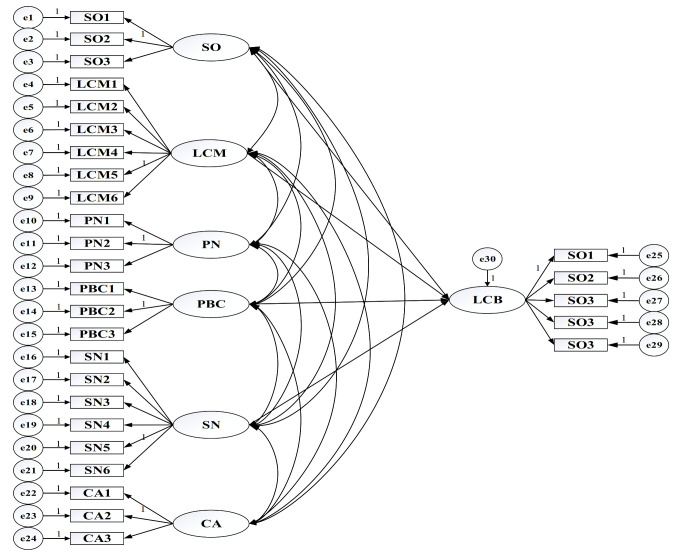
The initial structural model (M1).

The initial structural model (M1) was constructed using Amos 24 after the acceptable evaluation of the measurement model. The interactions of constructs (latent variables) and relevant items (observed variables) are listed in more detail (Figure 4). For effective model identification in confirmatory factor analysis, the unmeasured latent variables were applied to a set measurement scale and to capture the common latent factors by constraining the given paths equal to 1 [101], shown in Figure 4.

As shown in Table 10, there were two insignificant effect paths, i.e., CA→LCB and PN→LCB (*p* > 0.05) when the initial model was run—suggesting that the factors of customer attitude and personal norm exert no direct and significant effects on low-carbon behaviors in star-rated hotels. It can be found in Table 11 that this model was not perfect due to the goodness-of-fit index (GFI) being lower than the allowable limit (0.9). Hence, it is the current model that was necessarily required to be improved for better goodness-of-fit. The model evolved following the modification sequence from model reduction to model expansion in this research. Particularly, the model was modified each time by deleting the least significant path whose *p*-value was the highest in the duration of model reduction. Based on such guidelines, the paths of CA→LCB and PN→LCB were eliminated in an orderly fashion (*p* > 0.05), with M2 and M3 obtained in two modification rounds, respectively (Table 12). Still, the model fitness was not yet within a satisfying range after these deletions. Model expansion was then needed for further modification, as no more insignificant paths could be removed.

To improve scalar invariance, the modification index (MI) was used in this phase of model modification. The parameter with a high value of MI could be freed from constraints and this indicated the improvement of the given correlation [102]. Therefore, the model was thoroughly modified according to the largest MI for correlation improvement, with its practical significance considered. The new correlations were constructed successively as follows: “e18<=>e19” (MI = 66.049)→M4, “e17<=>e18” (MI = 34.907)→M5, “e16<=>e17” (MI = 23.244)→M6, “e3<=>e9” (MI = 20.802)→M7, “e28<=>e29” (MI = 17.051)→M8, “e4<=>e6” (MI = 15.903)→M9, leading to the final model being perfectly modified. Indices of model fitness in the whole process of model modification are presented in Table 12. The model would not continue to be modified until all the indices met their own standardized minimum thresholds.

#### 5.3.2. Assessment of the Tested Structural Model

**Figure 5:**
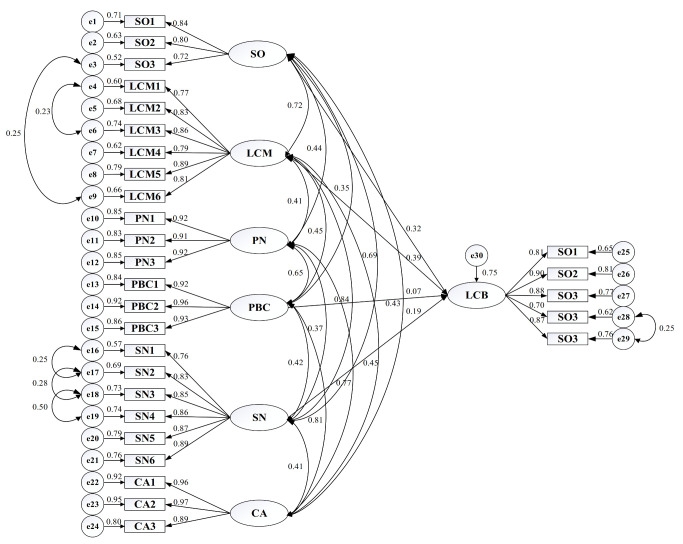
Standardized estimation of the final model (M9).

As shown in Table 12, all the goodness-of-fit indices in the modified model (M9) satisfied their own threshold values, which means that M9 had strong explanatory power and could be thus confirmed as the final model. Standardized estimation coefficients of the final model, as depicted on each path in Figure 5, reflects the weight that construct items contribute to latent variables and the impact of the influencing factors on low-carbon behaviors of staff in star-rated hotels. The standardized weights of different dimensions for construct items are more than 0.700 (*p* < 0.001), illustrating the fact that all of them significantly affect their corresponding constructs.

Th results of all hypothesized paths tested in the final model are presented in Table 13. Both PN and CA insignificantly affect low-carbon behaviors in star-rated hotels, indicating rejections of the original Hypothesis 3 and Hypothesis 6. LCM exerts the most positive 0.393 effect on low-carbon behaviors in star-rated hotels (*p* < 0.001), which supports Hypothesis 2, followed by SO, SN, and PBC, respectively. SO has a positive 0.322 effect (*p* < 0.001), supporting Hypothesis 1, and SN has a positive 0.192 effect (*p* < 0.01), supporting Hypothesis 5. By comparison, PBC seems to have the least influence, with a path coefficient of 0.074 (*p* < 0.05), which supports Hypothesis 4. The total and partial effects of the factors that influence low-carbon behaviors in star-rated hotels are shown in Table 14. More details of them will be discussed in the next section.

## 6. Discussion on Key Factors and Strategy Proposals

### 6.1. Key Factor 1: Low-Carbon Managerial Activities

The current results underline the priority of low-carbon managerial activities, indicating that low-carbon publicity (LCM5) stands out as the most crucial managerial solution affecting the implementation of staff’s low-carbon behaviors in star-rated hotels, whose direct influencing coefficient is the highest at 0.89. This is consistent with the orientation to low-carbon tourism consumption publicity proposed by Luo and Zhang (2011) [103], but the finding in this research reveal that public service announcements to hotel guests like low-carbon banners, signs, flags or other visuals would also guide the voluntary low-carbon behavior of internal staff by osmosis. Therefore, to ensure the effectivity of low-carbon management in star-rated hotels, soft solutions, such as low-carbon consumption publicity, green training, or organizing frequent voluntary activities designed to advance employees’ low-carbon operation experience, are all of necessity as education/learning functions to guide the low-carbon behavior adoption of staff. Simultaneously, as revealed in prior research [9,104], hard solutions cannot be omitted in management processes, e.g., low-carbon technology improvement or facility upgrades and appropriate rewards or penalties for staff from time to time. From the above, Strategy 1 is proposed to facilitate the low-carbon behaviors of staff working for star-rated hotels: put effective low-carbon management into practice and implement multiple managerial activities with both hard and soft approaches.

### 6.2. Key Factor 2: Strategic Orientation

Low-carbon strategic orientation is the second factor strongly affecting staff’s low-carbon behaviors in star-rated hotels, with a direct effect coefficient of 0.322. This is consistent with the findings of Bonilla et al. (2011) where the pro-environmental behaviors of most hotels they surveyed were internally driven by the purpose of the strategic decisions made by management [105]. According to the above results, the SO1 item suggests that hotel leadership is an important agent and must make low-carbon strategic orientation its duty, which is a prerequisite for hotel staff to follow the strategic objective of low-carbon hotel development. Moreover, the SO2 item indicates that low-carbon strategic orientation helps star-rated hotels build an eco-culture and a positive brand image to increase the advantages of gains in the market. These important invisible and priceless assets could exert a long-term subtle influence on hotel staff so as to lead them towards voluntarily regulating their own behaviors. Additionally, the SO3 item implies that it is a valuable approach for low-carbon strategic orientation in hotels by formulating explicit management regulations related to low carbon and energy conservation. Hence, Strategy 2 is then developed: improve the levels of low-carbon behavior by hotel staff, in order to enable a low-carbon strategic orientation that depends on strong leadership, culture/brand and management regulations, and create a benchmark in the lodging industry.

### 6.3. Key Factor 3: Social Norms

Social norm is the only external predictor with a direct significant impact of 0.192 on staff’s low-carbon behaviors in star-rated hotels. The findings are in line with the conclusion of Chen and Li (2019)—that governmental departments or non-governmental organizations should properly encourage and guide individual low-carbon behaviors through laws, policies and social media [84]. Social norms could be regarded as externalizations of personal ethics, showing the concrete reflection and embodiment of individual values in a certain context. There are two means by which to make full use of social norms from the results of this research. The first way is to create an inspired low-carbon atmosphere. In particular, in the mobile internet era, with high public awareness of low carbon and environmental protection, any high-carbon behavior by staff might be exposed, which urges star-rated hotels to consciously shoulder their due social responsibilities in order to avoid negative public opinions from mass media. Moreover, a healthy competitive micro-environment catalyzes low-carbon development in the hotel industry. Domestic star-rated hotels always compete closely with each other for limited market resources; meanwhile, they resist the competition pressure from international hotels, e.g., Marriott, Hilton, Accor, and Shangri-La. Under such conditions, the low-carbon behavior implementation of internal staff could help enhance hotel marketing competitiveness by controlling running costs and increasing the hotel’s social reputation. The second way to achieve this is by taking proper advantage of restraint-oriented social norms. This provides strong evidence for the view that governments play a specific, crucially important role [106]. If the government conducts administrative interventions, clarifies the demand for low-carbon principles, and provides the required market policies, low-carbon behavior among staff in the lodging industry will be constrained into a relatively normative pattern. Thus, Strategy 3 is proposed: encourage an inspiration-oriented social atmosphere along with a healthy competitive market environment and guide the timely implementation of restraint-oriented low-carbon policies.

### 6.4. Key Factor 4: Perceived Behavior Control

Perceived behavior control is another key factor affecting staff’s low-carbon behaviors in star-rated hotels (with the direct effect of 0.074). The findings that individual time and energy (PBC2) positively affect the low-carbon behaviors in star-rated hotels are in agreement with those proposed by Stone and Fernandez (2008) [107]. Based on this, more low-carbon education programs could be introduced for boosting staff behavior, in which they would be provided with more time and energy for reflection and participation, hence conducting targeted low-carbon behaviors [108,109,110]. It was previously found that environmental knowledge, reflecting personal literacy, influenced employees’ associated attitudes and behaviors [111]. Our findings further support this result that knowledge level (PBC1) is particularly crucial to low-carbon behavior adoption for star-rated hotels. Furthermore, the hypothesis of the positive effect of individual competitiveness (PBC3) on staff’s low-carbon behaviors is proven. As for a star-rated hotel that is used to implementing low-carbon practices, hotel managers prefer to select employees with more energy and carbon reduction knowledge than those with less. Therefore, it seems that a high low-carbon knowledge level helps increase the individual competitiveness of employees and the possibility of gaining access to work or even promotion. Moreover, in agreement with the view of Teng et al., (2014) [111], the hotel industry is expected to carry out motivation and incentive programs for energy conservation and carbon reduction in order to make their staff more competitive by applying low-carbon knowledge and developing pro-environmental awareness and behaviors. Therefore, Strategy 4 is accordingly proposed: frequently provide comprehensive education and incentive programs for hotel staff at different levels in order to enhance the ability of their perceived behavior control.

## 7. Conclusions

Guiding sustainable development across the lodging industry normally requires the support and involvement of hotel staff, so the implementation of low-carbon behaviors among these staff can make a great difference in helping star-rated hotels become green. Therefore, this research aimed to identify the key influencing factors of staff’s low-carbon behaviors in star-rated hotels and their respective impact both inside and outside of the hotel context. First, a set of six influencing factors of low-carbon behavior among staff was identified according to literature retrieval, grounded theory and in-depth interviews, presented as a hypothesized model for triggering low-carbon behavior among staff in star-rated hotels. Structural equation modelling (SEM) was then applied to explore the impact of the identified factors on low-carbon behavior among staff implementation in star-rated hotels. Based on the study findings and discussions provided above, targeted strategies were then proposed.

The results show that three internal factors (i.e., strategic orientation, low-carbon managerial activities, and perceived behavior control) and one single external factor (i.e., social norms) significantly affected hotel staff’s low-carbon behaviors. More specifically, the factor of low-carbon managerial activities exerts the most critical influencing factor. However, personal norms and consumer attitude were found to have no statistically significant effect on hotel staff’s low-carbon behaviors. Finally, four strategies were discussed in relation to how to facilitate low-carbon practices by staff on the basis of key factor analyses: Strategy 1—put effective low-carbon management into practice and implement multiple managerial activities with both hard and soft approaches; Strategy 2—improve the levels of low-carbon behavior by hotel staff, in order to enable a low-carbon strategic orientation that depends on strong leadership, culture/brand and management regulations, and create a benchmark in the lodging industry; Strategy 3—encourage an inspiration-oriented social atmosphere along with a healthy competitive market environment and guide the timely implementation of restraint-oriented low-carbon policies; and Strategy 4—frequently provide comprehensive education and incentive programs for hotel staff at different levels in order to enhance the ability of their perceived behavior control.

The findings in this research offer both theoretical implications and practical implications. Generally, prior studies on the influencing factors of low-carbon behavior in the hospitality industry mainly stressed what triggered the low-carbon behavior of hotel guests rather than that of the hotel service side. Some others conducted studies only on individual psychological levels, focusing on psychology-related factors that affected the behavior of either managers or employees in hotels. Hence, this research makes up for the insufficient literature by identifying the influencing factors of hotel staff’s low-carbon behaviors both inside and outside of the hotel context. Although the factor of consumer attitude had a negative impact in this research, it could lead to further academic discussions about the relationship between hotel consumers and low-carbon behavior adoption in the hospitality industry. The findings also present several practical implications. Despite legislation tightening low-carbon policies and prioritizing eco-operation in low-carbon industries, a comprehensive system of policies and norms regarding low-carbon development is still lacking in the lodging sector nationwide. Hence, the current research is conducive to improving relevant policies and norms and for the authorities to close the gap between low-carbon hotel practice and policymaking. Furthermore, the proposed strategies provide a reference for hoteliers and the government on how to guide the effective implementation of low-carbon behaviors among hotel staff, which not only helps to create a low-carbon competitive hospitality market, but also contributes to conveying pro-environmental attributes to the whole of society. Although this is an empirical study of Eastern China, the research findings could be used in other regions or countries.

The research aim has been successfully achieved, though there are still some limitations. The present research was limited to different star-rated hotels for homogeneity, while future work will focus on the differences among hotels with different stars. Moreover, this empirical survey was restrictedly conducted in typical star-rated hotels in Eastern China. Larger samples from other, different areas will be collected and theoretical models will be optimized for confirmatory factor analysis in further research. Meanwhile, hotel differences caused by whether or not they belong to a hotel chain will be also considered in the future in order to explore the influence of chain management pressure on the low-carbon behaviors of staff.

## Figures and Tables

**Table 1:** Low-carbon behaviors of staff in star-rated hotels.

Category	Behavior
Low-carbon behaviors of hotel staff	low-carbon design behavior
	low-carbon procurement behavior
	low-carbon decision-making behavior
	low-carbon operation behavior
	low-carbon execution behavior

**Table 2:** Identification of factors influencing low-carbon staff behavior in star-rated hotels.

Category	Factors	Existing Variables
Internal factors	Strategic orientation	Corporate social responsibility
		Low-carbon corporate culture
		Low-carbon corporate image
		Proactive environmental strategy
		Top management support
	Low-carbon managerial activities	System of rewards and penalties
		Available resources for implementation
		Green training
		Disposal of throw-away products
		Low-carbon publicity
		Communication and interactions
	Personal norms	Individual green values
		Environmental attitude
		Pro-environmental reputation
		Environmental will and initiatives
	Perceived behavior control	Low-carbon knowledge
		Time and energy
		Individual self-competitiveness
External factors	Social norms	Marketing policy
		Laws, standards and regulations
		Government supervision
		Mess media
		Nongovernmental organization supervision
		Pressure from peer hotels
	Consumer attitude	Willingness to cooperate with low-carbon behavior
		Demanding sustainable products
		Check-in satisfaction and loyalty
		Intention towards green hotel visit

**Table 3:** Factors and measurement items for low-carbon staff behavior adoption in star-rated hotels.

Latent Factors	Code	Items for Construct
Strategic orientation (SO)	SO1	Hotel leadership places an emphasis on low carbon/environmental protection and considers both as being part of a social mission.
	SO2	Hotels have a low-carbon enterprise culture and brand image that are praised.
	SO3	Hotels formulate their own low-carbon management regulations.
Low-carbon managerial activities (LCM)	LCM1	Hoteliers provide rewards or penalty to employees according to their performance in relation to low-carbon practices.
	LCM2	Low energy-efficiency facilities are regularly phased out, and energy-saving reforms are actively conducted with new technologies.
	LCM3	Regular training is provided for hotel staff in order to popularize knowledge of low-carbon practices in hotels.
	LCM4	Disposable toiletries (e.g., prepackaged toothbrushes, toothpaste, shampoo, soap, combs, and slippers) are slimmed down, with reusable ones provided instead.
	LCM5	Low-carbon publicity activities are frequently carried out, such as putting up banners or slogans about environmental protection on the walls and tip cards with energy-saving reminders in guests’ rooms.
	LCM6	Hoteliers organize or participate in frequent voluntary activities regarding low-carbon development for communication and interactions with other peer enterprises.
Personal norms (PN)	PN1	Staff in hotels assume social responsibility for emission reductions and environmental protection.
	PN2	Staff in hotels would like to boost their personal reputations and relationship with others through low-carbon behavior implementation.
	PN3	Staff in hotels show great willingness towards low-carbon practices and environmental protection.
Perceived behavior control (PBC)	PBC1	Staff in hotels have a high-level knowledge and understanding of reducing CO_2_ emissions.
	PBC2	Staff in hotels have adequate time and energy to practice low-carbon behaviors.
	PBC3	Staff in hotels face great demand to enhance their own competitiveness through low-carbon behavior implementation.
Social norms (SN)	SN1	Macro-level market policies (e.g., energy price guidance, ladder-type electricity pricing, etc.) obviously influence low-carbon practice in hotels.
	SN2	Norms and standards regarding hotel management provide effective references for low-carbon practice.
	SN3	Supervision from social administrative departments such as environmental protection agencies leads hotels towards low-carbon behavior implementation for social sustainability.
	SN4	Public opinion from mass media makes hotels move towards low-carbon behavior implementation for social sustainability.
	SN5	There is a low-carbon environmental protection atmosphere throughout the whole of society, with the focus increasing on low-carbon practices in the hotel industry.
	SN6	Peer competitiveness brings so much pressure to hotels that low-carbon practice becomes an indispensable part.
Consumer attitude (CA) ^1^	CA1	Consumers support the low-carbon behavior of hotels and are willing to cooperate with them.
	CA2	Consumers have great demand for low-carbon/green products in hotels.
	CA3	Consumers always offer their comments to improve the low-carbon/green behavior implementation in hotels.
Low-carbon behavior (LCB)	LCB1	Operations managers get involved in the design of low-carbon strategies such as those related to water saving, renewable use, operating facilities, etc.
	LCB2	Low-carbon green products are the first to be purchased in the procurement process.
	LCB3	Investment in low-carbon practice has increased due to decision making.
	LCB4	Hoteliers implement low-carbon strategies into the whole process of operational management.
	LCB5	Employees perform their duties by actively executing low-carbon practices at work.

^1^ As consumer attitude (CA) was identified as an external factor of the low-carbon behavior of hotel staff, it was measured from the hotel staff’s point of view, i.e., the impact of customer attitude on low-carbon staff behavior was rated by staff.

**Table 4:** Summary of respondent socio-demography (*n* = 440).

Characteristics		*n*	Proportion (%)
Gender	Male	251	57.0%
	Female	189	43.0%
Age	<25	62	14.2%
	25–30	91	20.8%
	31–40	160	36.5%
	>40	125	28.5%
Education level	Senior school or below	131	29.8%
	Junior college	174	39.5%
	College	126	28.6%
	Master or above	9	2.1%
Occupation in hotel	Service staff member	122	27.7%
	Department manager	249	56.6%
	Chief inspector	47	10.7%
	Managing director or above	22	5.0%
Working experience in hotel industry	<5 years	144	32.7%
	5–10 years	145	33.0%
	11–20 years	116	26.4%
	>20 years	35	7.9%
Number of hotels with different stars	Three star-rated hotels	131	29.8%
	Four star-rated hotels	189	43.0%
	Five star-rated hotels	120	27.2%

**Table 5:** Descriptive statistics of construct items.

Construct Item	Overall Sample (*n* = 440)			
	M	SD	*Sk*	*K*
SO1	6.18	0.987	−1.032	0.834
SO2	6.33	0.855	−1.149	1.141
SO3	6.45	0.839	−1.901	5.599
LCM1	5.97	1.265	−1.129	0.668
LCM2	6.20	1.034	−1.348	1.199
LCM3	6.07	1.196	−1.207	0.657
LCM4	6.04	1.237	−1.046	0.205
LCM5	6.12	1.138	−1.198	0.480
LCM6	6.23	1.108	−1.417	1.234
PN1	5.75	1.065	−0.480	−0.558
PN2	5.78	1.034	−0.386	−0.762
PN3	5.74	1.085	−0.487	−0.561
PBC1	5.86	1.017	−0.651	−0.125
PBC2	5.74	1.111	−0.550	−0.579
PBC3	5.70	1.159	−0.641	−0.418
SN1	6.45	0.828	−1.545	1.993
SN2	6.31	0.930	−1.364	1.423
SN3	6.30	1.020	−1.512	1.661
SN4	6.24	1.076	−1.457	1.344
SN5	6.12	1.197	−1.225	0.801
SN6	6.18	1.117	−1.279	1.285
CA1	5.78	1.111	−0.622	−0.245
CA2	5.77	1.149	−0.697	−0.234
CA3	5.63	1.173	−0.559	−0.371
LCB1	6.37	0.974	−1.506	2.074
LCB2	6.25	1.020	−1.400	1.592
LCB3	6.29	1.043	−1.409	1.350
LCB4	6.42	1.002	−2.012	4.329
LCB5	6.32	1.043	−1.723	2.990

Note: M = mean; SD = standard deviation; *Sk* = skewness value; *K* = kurtosis value. SO = strategic orientation; LCM = low-carbon managerial activities; PN = personal norms; PBC = perceived behavior control; SN = social norms; CA = consumer attitude; LCB = low-carbon behavior.

**Table 6:** Results of the reliability test.

Construct	Cronbach’s α ^a^	CR	No. of Items
Strategic orientation (SO)	0.820	0.826	3
Low-carbon managerial activities (LCM)	0.928	0.929	6
Personal norms (PN)	0.942	0.942	3
Perceived behavior control (PBC)	0.953	0.954	3
Social norms (SN)	0.940	0.943	6
Consumer attitude (CA)	0.958	0.960	3
Low-carbon behavior (LCB)	0.930	0.930	5

Note: CR = composite reliability. ^a^ Overall Cronbach’s α = 0.963.

**Table 7:** Rotated component matrix.

Construct Item	Components					
	1	2	3	4	5	6
SO1	0.773	-	-	-	-	-
SO2	0.780	-	-	-	-	-
SO3	0.665	-	-	-	-	-
LCM1	-	0.788	-	-	-	-
LCM2	-	0.681	-	-	-	-
LCM3	-	0.771	-	-	-	-
LCM4	-	0.730	-	-	-	-
LCM5	-	0.709	-	-	-	-
LCM6	-	0.655	-	-	-	-
PN1	-	-	0.853	-	-	-
PN2	-	-	0.858	-	-	-
PN3	-	-	0.842	-	-	-
PBC1	-	-	-	0.887	-	-
PBC2	-	-	-	0.905	-	-
PBC3	-	-	-	0.870	-	-
SN1	-	-	-	-	0.713	-
SN2	-	-	-	-	0.789	-
SN3	-	-	-	-	0.839	-
SN4	-	-	-	-	0.857	-
SN5	-	-	-	-	0.781	-
SN6	-	-	-	-	0.688	-
CA1	-	-	-	-	-	0.628
CA2	-	-	-	-	-	0.649
CA3	-	-	-	-	-	0.616

Note: Extraction method: Principal Component Analysis (PCA); Rotation: varimax rotation standardized by Kaiser (rotation is convergent after the eighth iteration); cumulative variance contribution: 82.01%; Kaiser-Meyer-Olkin (KMO) statistic: 0.942 (very acceptable); Bartlett’s Test of Sphericity probability: 0.000.

**Table 8:** Converged validity and goodness-of-fit of the measurement model.

Construct			Item				FL			AVE			
SO										0.614			
			SO1				0.835 ***						
			SO2				0.793 ***						
			SO3				0.718 ***						
LCM										0.687			
			LCM1				0.789 ***						
			LCM2				0.828 ***						
			LCM3				0.870 ***						
			LCM4				0.785 ***						
			LCM5				0.884 ***						
			LCM6				0.813 ***						
PN										0.845			
			PN1				0.921 ***						
			PN2				0.912 ***						
			PN3				0.924 ***						
PBC										0.874			
			PBC1				0.916 ***						
			PBC2				0.958 ***						
			PBC3				0.930 ***						
SN										0.735			
			SN1				0.760 ***						
			SN2				0.864 ***						
			SN3				0.904 ***						
			SN4				0.903 ***						
			SN5				0.866 ***						
			SN6				0.838 ***						
CA										0.888			
			CA1				0.958 ***						
			CA2				0.972 ***						
			CA3				0.895 ***						
LCB										0.728			
			LCB1				0.801 ***						
			LCB2				0.891 ***						
			LCB3				0.876 ***						
			LCB4				0.810 ***						
			LCB5				0.884 ***						
X^2^/df	*p*	GFI		AGFI	RMSEA	TLI		IFI	CFI		PNFI	PGFI	
2.557	0.000	0.872		0.843	0.060	0.952		0.958	0.958		0.818	0.713	

Note: FL = standardized factor loading; AVE = average variance extracted; GFI = goodness-of-fit index; AGFI = adjusted goodness-of-fit index; RMSEA = root mean square error of approximation; TLI = Tucker–Lewis Index; IFI = incremental fit index; CFI = comparative fit index; PNFI = parsimony normed-fit index; PGFI = parsimony goodness-of-fit index. *** *p* < 0.001.

**Table 9:** Correlation matrix and discriminant validity for the constructs.

Construct	SO	LCM	PN	PBC	SN	CA	LCB
SO	(0.783)	-	-	-	-	-	-
LCM	0.724 ***	(0.829)	-	-	-	-	-
PN	0.450 ***	0.416 ***	(0.919)	-	-	-	-
PBC	0.349 ***	0.449 ***	0.655 ***	(0.935)	-	-	-
SN	0.672 ***	0.817 ***	0.359 ***	0.412 ***	(0.857)	-	-
CA	0.440 ***	0.448 ***	0.774 ***	0.811 ***	0.402 ***	(0.942)	-
LCB	0.768 ***	0.815 ***	0.367 ***	0.447 ***	0.754 ***	0.413 ***	(0.853)

Note: Bracketed values are the square roots of the average variance extracted. *** *p* < 0.001.

**Table 10:** Regression weights in the initial model (M1).

Path			Estimate	SE	*t*-Statistic	*p*-Value
SO	→	LCB	0.500	0.078	6.436	***
LCM	→	LCB	0.339	0.059	5.738	***
PN	→	LCB	−0.069	0.040	−1.751	0.080
PBC	→	LCB	0.149	0.041	3.670	***
SN	→	LCB	0.133	0.049	2.733	**
CA	→	LCB	−0.071	0.049	−1.453	0.146

Note: *** *p* < 0.001. ** *p* < 0.01.

**Table 11:** Goodness-of-fit of the initial structural model (M1).

Goodness-of-Fit Measure		Level of Acceptance Fit	Fit Statistics
Absolute fit	X^2^/df	<3.00	2.572
	GFI	>0.90	0.871
	AGFI	>0.80	0.843
	RMSEA	<0.08	0.060
Incremental fit	TLI	>0.95	0.951
	IFI	>0.90	0.957
	CFI	>0.90	0.957
Parsimonious fit	PNFI	>0.50	0.822
	PGFI	>0.50	0.844

**Table 12:** Goodness-of-fit in the process of model modification.

Goodness-of-Fit Measure		Fit Statistics								Level of Acceptance Fit
		M2	M3	M4	M5	M6	M7	M8	M9	
Absolute fit	X^2^/df	2.555	2.572	2.340	2.242	2.178	2.124	2.074	2.030	<3.00
	GFI	0.871	0.871	0.881	0.886	0.889	0.893	0.897	0.901	>0.90
	AGFI	0.843	0.843	0.855	0.860	0.864	0.869	0.873	0.876	>0.80
	RMSEA	0.060	0.060	0.055	0.053	0.052	0.051	0.049	0.048	<0.08
Incremental fit	TLI	0.952	0.951	0.958	0.962	0.963	0.965	0.967	0.968	>0.95
	CFI	0.958	0.957	0.963	0.966	0.968	0.970	0.971	0.972	>0.90
	IFI	0.958	0.957	0.964	0.966	0.968	0.970	0.971	0.973	>0.90
Parsimonious fit	PNFI	0.820	0.822	0.825	0.825	0.824	0.823	0.822	0.821	>0.50
	PGFI	0.842	0.844	0.847	0.847	0.846	0.845	0.844	0.843	>0.50

**Table 13:** Results of hypothesis test in the final model.

Path			Hypothesis	Coefficient	*t*-Statistic	*p*-Value
SO	→	LCB	H1	0.322	6.102	***
LCM	→	LCB	H2	0.393	5.355	***
PBC	→	LCB	H4	0.074	2.175	*
SN	→	LCB	H5	0.192	2.996	**

Note: *** *p* < 0.001. ** *p* < 0.01. * *p* < 0.05.

**Table 14:** Effects of factors on low-carbon behavior of staff in hotels (LCB) in the final model.

Effects on LCB	SO	LCM	PBC	SN
LCB	0.322	0.393	0.074	0.192
LCB1	(0.259)	(0.316)	(0.059)	(0.155)
LCB2	(0.290)	(0.353)	(0.066)	(0.173)
LCB3	(0.283)	(0.345)	(0.065)	(0.169)
LCB4	(0.253)	(0.309)	(0.058)	(0.151)
LCB5	(0.280)	(0.341)	(0.064)	(0.167)

Note: Values in the first line indicate the total effects of each influencing factor on LCB. Bracketed values in the following lines indicate the partial effect on each construct item of LCB. PN and CA have no influence on LCB, so they are not shown.
